# High-grade astrocytoma with piloid features: a single-institution case series and literature review

**DOI:** 10.1186/s40478-025-01987-0

**Published:** 2025-04-24

**Authors:** Eric A. Goethe, Subhiksha Srinivasan, Swaminathan Kumar, Sujit S. Prabhu, Maria A. Gubbiotti, Sherise D. Ferguson

**Affiliations:** 1 Department of Neurosurgery, University of Texas MD Anderson Cancer Center, Houston, TX 77030 USA; 2 Department of Neurosurgery, Baylor College of Medicine, Houston, TX 77030 USA; 3 Department of Neurosurgery, McGovern Medical School, Houston, TX 77030 USA; 4 Department of Melanoma Medical Oncology, University of Texas MD Anderson Cancer Center, Houston, TX 77030 USA; 5 The University of Texas MD Anderson Cancer Center UTHealth Houston Graduate School of Biomedical Sciences, Houston, TX 77030 USA; 6 Division of Pathology and Laboratory Medicine, University of Texas MD Anderson Cancer Center, Houston, TX 77030 USA

**Keywords:** Atypical glioma, Methylation profiling, Astrocytoma, BRAF mutation

## Abstract

High-grade astrocytoma with piloid features (HGAP) is a recently described primary brain tumor and the first requiring a specific methylation pattern for diagnosis, as its histologic features are often compatible with other tumors such as glioblastoma (GBM). Characterized by molecular alterations in *CDKN2A/B*,* NF1*,* BRAF*,* FGFR1*, and *ATRX*, they may be located anywhere in the CNS but show a predilection for the posterior fossa. Reports are limited to retrospective case series, and the standard of care is not yet established. We performed a retrospective review of electronic medical records of all patients with HGAP at our institution. Records were queried for demographic, radiographic, clinical, surgical, pathologic, and outcome data. Eighteen patients were included with a median 17.1 months follow-up. Of these, 12 (63.2%) were women with a mean age of 43 years (range 24–67). The most common tumor locations were the cerebellum (8 patients, 42.1%) and thalamus (6 patients, 31.6%). On imaging, tumors were most commonly homogeneously contrast-enhancing (10 patients, 52.6%) or rim enhancing with central necrosis (5 patients, 26.3%). Ten patients (52.6%) underwent biopsy, while nine (47.4%) underwent resection, of which four (44.4%) underwent gross total resection. Adjuvant therapy included radiation in 16 patients (88.9%) and systemic treatment in 16 patients (88.9%). The initial systemic treatment was temozolomide in 14 patients (77.8%). One patient received upfront trametinib (a MEK1 inhibitor), and one patient received upfront dabrafenib (a BRAF inhibitor). At last follow up, 11 patients (57.9%) had progressive disease. Median progression-free survival (PFS) was 5.4 months (range 1.6–28.2 months), and median overall survival (OS) had not been reached. HGAP is a newly described rare glial tumor without an established standard of care. Its aggressive behavior and targetable mutations warrant further investigation regarding predictors of outcome for this entity.

## Introduction

High-grade astrocytoma with piloid features (HGAP) is a recently described primary glioma and the first glioma requiring a specific methylation pattern for diagnosis [2, 10]. HGAP was initially described by Reinhardt in 2018, appearing early in the fifth decade of life and characterized by molecular alterations in *CDKN2A/B*, *NF1*, *BRAF*, *FGFR1*, and *ATRX* [2, 4, 11]. This lesion may be located anywhere in the central nervous system (CNS) but shows a predilection for the posterior fossa [4]. Histologically, such neoplasms can show microvascular proliferation or necrosis akin to glioblastoma (GBM) and, though most are circumscribed, some can show either focal or more prominent infiltrative growth patterns [4]. While this entity was included in the 2021 WHO Classification for CNS Tumors, its management is not yet standardized [4, 6]. Multiple treatment regimens have been described, including standard of care chemoradiation followed by adjuvant temozolomide, or targeted agents such as BRAF or MEK inhibitors [2, 11]. In order to further characterize this entity, we performed a retrospective chart review of all cases of HGAP at a single institution.

## Methods

We performed a retrospective review of electronic medical records of all patients diagnosed with HGAP at our institution. Search criteria included the diagnosis “high grade astrocytoma with piloid features” or “HGAP.” Other search criteria included “posterior fossa glioma,” “glioma with piloid features,” and “atypical pilocytic astrocytoma.” Records were queried for demographic, radiographic, clinical, surgical, pathologic, DNA methylation profiling, and outcome data. Demographic data included age at time of surgery, sex, race, and other cancer history. Radiographic data included tumor location and size. Clinical data included presenting symptoms, presence of seizures, performance status as measured by the Karnofsky Performance Score (KPS), and the use of systemic and/or radiation therapy. Surgical data included type of surgery (classified as biopsy, subtotal resection, or gross total resection), length of stay after surgery, and discharge disposition. Pathologic data included the characterization of various mutations and genomic alterations which included all standard glioma-associated genes including the *TERT* promoter. All pathology was reviewed by a board-certified neuropathologist. Outcome data included progression-free survival (defined as the interval between the date of surgery and radiographic progression) and overall survival (defined as the interval between the date of pathological diagnosis/surgery and death)(see Table 1).

## Results

**Fig. 1:**
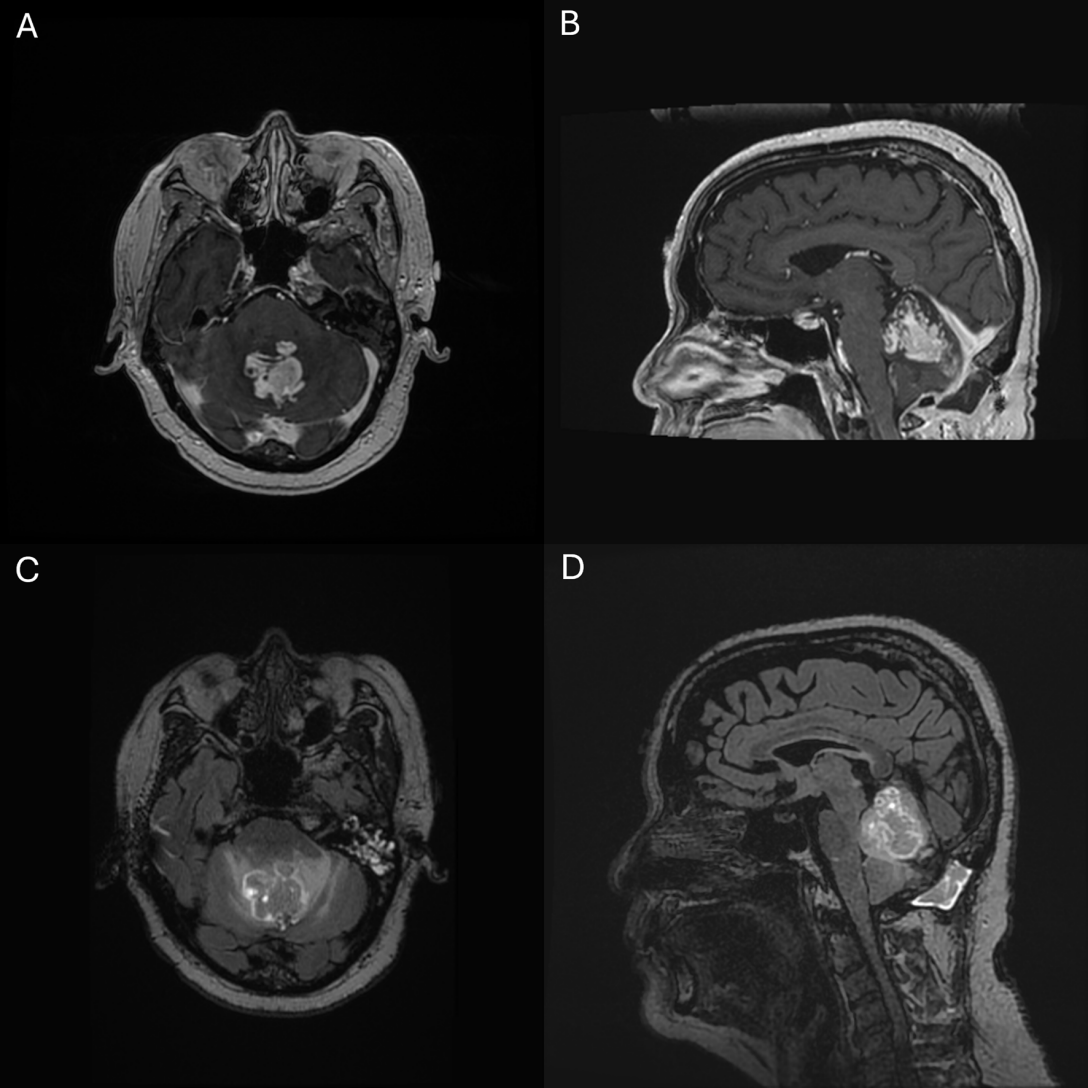
T1 post-contrast (**A**) axial and (**B**) sagittal and T2 FLAIR (**C**) axial and (**D**) sagittal magnetic resonance images of vermian HGAP in a 68-year-old man. The mass and surrounding cerebellar folia display marked enhancement, suggesting leptomeningeal spread

Patient data are presented in Table 1. There were 18 patients with HGAP identified at our institution with a median 17.1 months follow-up after pathologic diagnosis. Eleven patients (61.1%) were women. The mean age was 44.4 years (SD 12.5, range 24–67). The mean preoperative KPS was 80 (SD 9.4, range 70–100). Three patients (16.7%) had a history of Neurofibromatosis Type 1 (NF1). The most common tumor locations were cerebellar (seven patients, 38.9%) and thalamic (six patients, 33.3%). On imaging, tumors were most commonly homogenously contrast-enhancing (ten patients, 55.5%), followed by rim-enhancing with central necrosis (five patients, 27.8%) and patchy enhancement (three patients, 16.7%) (Fig. 1). Six patients (31.6%) had extensive peritumoral FLAIR attenuation suggestive of invasive disease. Three patients (of 16 with available data) had diffusion restriction, though all were patchy or mild. Radiographic leptomeningeal infiltration was present at diagnosis in three patients (16.7%). Nine patients (50.0%) underwent biopsy for diagnostic confirmation, while nine (50.0%) underwent resection, of which four (44.4%) underwent gross total resection. Three patients (33.3%) who underwent resection had preceding biopsies to determine treatment plans. Following surgery, patients remained in the hospital for a mean of 4.0 days (range 0–14), and four (33.3%) were discharged to an inpatient rehabilitation facility. The mean KPS at discharge was 90 (SD 7.9, range 70–100).

**Table 1:** Institutional data

Patient	Age/ Sex^1^	Pathology	Tumor location	Surgery^2^	Postoperative KPS^3^	Radiation dose (cGy)	Systemic treatment	Progression-free survival (months)	Treatment for progression	Overall survival (months)
1	42 F	ATRX loss and mutation; CDKN2A loss, KIAA1549::BRAF and PTPRZ1::MET; MGMT promoter status unknown	Cerebellum	STR	90	n/a	n/a	1.57	Resection, irradiation	52.77
2	38 F	CDKN2A/B loss, ATRX mutation	Temporal	Biopsy-> GTR		52	Concurrent TMZ^4^	16.03	Resection, avastin, optune	LTFU
3	36 M	ATRX expression retained but mutation detected; NF1 mutation; MGMT promoter status unknown	Temporal	Biopsy	90	Unk	Concurrent TMZ + adjuvant TMZ	LTFU	LTFU	LTFU
4	24 F	ATRX loss of expression and mutation; CDKN2A/B loss; NF1 mutation, MGMT promoter unmethylated	Thalamus	Biopsy	90	46	Concurrent TMZ + adjuvant TMZ	10.03	Reirradiation	15.60
5	31 F	ATRX loss of expression and mutation; CDKN2A/B loss, NF1 mutation and subclonal BRAF G494V mutation; MGMT promoter status indeterminate	Thalamus	Biopsy-> STR	80	60	Concurrent TMZ + adjuvant TMZ	3.43	Re-irradiation, selumetanib	LTFU
6	47 F	ATRX mutation; CDKN2A loss, FGFR1 mutation, MGMT promoter status indeterminate	Thalamus	Biopsy		Unk	Concurrent TMZ + adjuvant TMZ	5.40	Avastin	LTFU
7	52 F	ATRX mutation; CDKN2A/B loss; MGMT status unknown	Thalamus	Biopsy		54	Concurrent TMZ + adjuvant TMZ	n/a	n/a	n/a
8	62 F	ATRX loss of expression and mutation; FGFR1 mutation, amplification of CDK4 and MDM2, copy number loss of RB1; MGMT promoter unmethylated	Brainstem	GTR		39	Concurrent TMZ + adjuvant TMZ	n/a	n/a	n/a
9	27 M	CDKN2A/B loss, BRAF V600E, MGMT promoter unmethylated	Cerebellum	GTR	100	n/a	n/a	5.23	Radiation, Dabrafenib, trametinib	n/a
10	42 M	No ATRX mutation; CDKN2A/B loss, NF1 mutation; MGMT promoter methylated	Cerebellum	Biopsy		60	Concurrent TMZ	LTFU	n/a	LTFU
11	31 M	ATRX loss of expression; BRAF V600E; MGMT promoter status indeterminate	Temporal	STR	80	Unk	Concurrent TMZ + adjuvant TMZ, Optune	28.17	TMZ, LITT, Dabrafenib/trametinib, investigational BRAF inhibitor	LTFU
12	37 M	ATRX loss of expression and mutation; CDKN2A/B loss, KIAA1549::BRAF, TGFBR1 mutation; MGMT promoter methylated	Cerebellum	Biopsy	90	n/a	Trametinib	LTFU	LTFU	LTFU
13	53 F	ATRX loss of expression and mutation; CDKN2A/B loss, FGFR1 mutation, MGMT promoter methylated	Thalamus	Biopsy	90	n/a^5^	n/a^5^	n/a^5^	n/a	n/a
14	67 M	Retained ATRX expression; RB1 mutation; MGMT promoter methylated	Cerebellum	Biopsy-> STR	90	60	Adjuvant TMZ	n/a	n/a	n/a
15	59 F	ATRX loss of expression and mutation; CDK2NA/B loss, MGMT promoter unmethylated	Cerebellum	GTR		Unk	Concurrent TMZ + adjuvant TMZ	2.13	LTFU	LTFU
16	50 M	ATRX loss of expression and mutation, NF1 mutation, MGMT promoter unmethylated	Spinal Cord	STR	80	40	Adjuvant TMZ	7.73	Selumetinib	13.17
17	43 F	ATRX loss of expression, MGMT promoter unmethylated	Medulla	Biopsy	70	54	None	3.17	Avastin	n/a
18	58 F	Retained ATRX expression; CDKN2A loss, NF1 mutation, TP53 mutation, MGMT promoter unmethylated	Cerebellum	Biopsy	80	60	Concurrent TMZ	n/a	n/a	n/a

^1^Age in years at time of surgery; ^2^STR= subtotal resection, GTR = gross total resection; ^3^KPS=Karnofsky performance score; ^4^TMZ=temozolomide; ^5^Patient treated recently and has not yet begun treatment

**Fig. 2:**
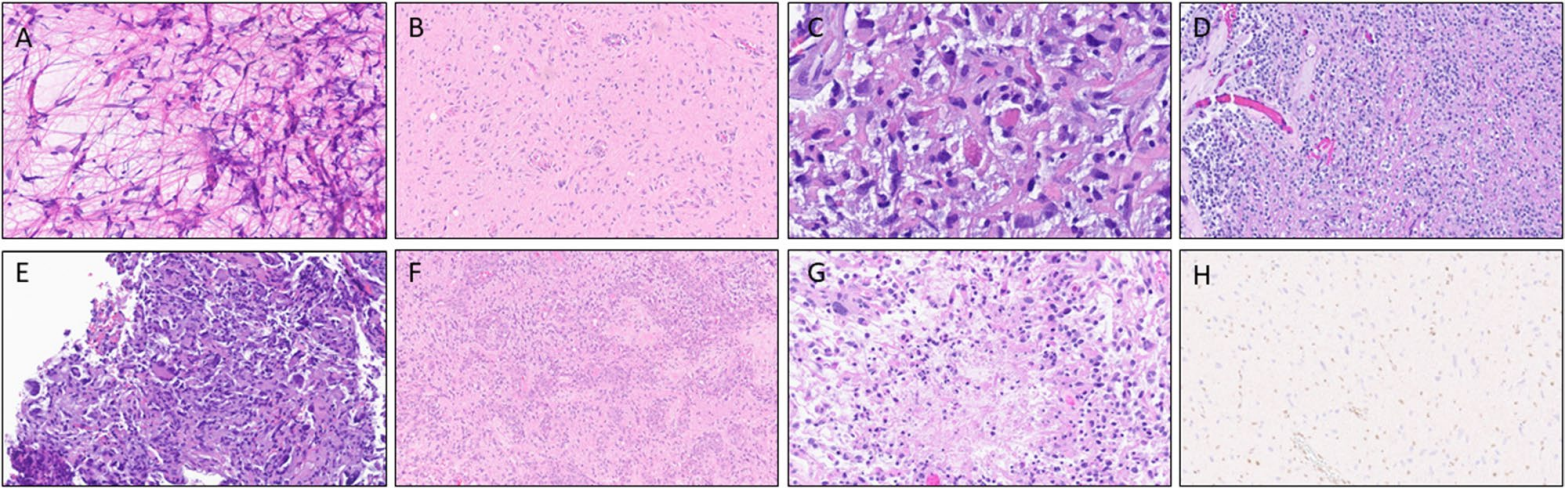
Microscopic findings of HGAP to demonstrate the variable morphology with (**A**) smear preparation showing long delicate piloid processes and admixed Rosenthal Fibers, (**B**) a mildly to moderately cellular region (**C**) higher power view of scattered eosinophlic granular bodies and Rosenthal fibers, (**D**) areas of oligodendroglial morphology, (**E**) foci demonstrating highly atpical cells with bizarre nuclear atypia, **F**), extensive microvascular/glomeruloid proliferation, **G**) foci of necrosis, and **H**) loss of nuclear expression of ATRX within the atypical tumor cells with appropriate internal positive control showing retained expression in endothelial cells

Histologic examination of the tumors showed variable morphology with some showing classical piloid features, some having an oligodendroglial appearance, many exhibiting prominent microvascular proliferation, few demonstrating focal necrosis, and others that were more densely cellular with highly atypical/multinucleated cells (Fig. 2A-G). All but one patient had molecular data available for review. Thirteen patients (72.2%) had either loss of protein expression of ATRX (Fig. 2H) or mutation in *ATRX*. Overall, 11 total patients (61.1%) demonstrated loss of *CDKN2A* and/or *CDKN2B*. Six patients (33.3%) had *NF1* mutations, three patients (16.7%) carried *FGFR1* mutations, two patients (11.1%) harbored the *KIAA1549::BRAF* fusion (one of which also harbored a concurrent *PTPRZ1::MET* rearrangement), and two patients (11.1%) demonstrated the *BRAFV600E* mutation. One patient showed a subclonal *BRAFG494V* alteration of uncertain clinical relevance. Only one patient demonstrated a pathogenic *TP53* mutation, and two patients showed *RB1* alterations (one with copy number loss and one with pathogenic mutation). While no *TERT* promoter mutations were identified in this cohort, one tumor harbored a *TERT* mutation p.G433D, which is of unknown significance. The *MGMT* promoter was methylated in only four out of fourteen cases with available data (22.2%). All but 5 cases underwent DNA methylation profiling. All cases matched to the class of “High Grade Astrocytoma with Piloid Features” except 2: one did not have an exact match, likely due to low tumor quantity, and a suggestive score for “high grade astrocytoma with piloid features” was noted; the second matched to “atypical pilocytic astrocytoma.” This latter case had DNA methylation profiling studies performed at a different institution than the other cases; this tumor class name was given by the institution and, based on the description in the outside report, reflects the class of “high grade astrocytoma with piloid features.”

Information regarding postoperative treatment was available for 17 patients. Adjuvant therapy included radiation in 15 patients (88.2%) and systemic treatment in 15 patients (88.2%). The initial systemic treatment was temozolomide in 13 patients (76.4%). One patient received upfront trametinib (a MEK1 inhibitor), and one patient received upfront dabrafenib (a BRAF inhibitor).

At the time of this writing, 11 patients (61.1%) had progressive disease. Median progression-free survival (PFS) was 5.0 months (SD 7.7, range 1.6 to 28.2 months). Median overall survival was 15.6 months (SD 22.2, range 13.2–52.8 months).

## Discussion

HGAP is a recently defined malignant primary brain tumor without an established standard of care. Earlier reports of HGAP arose from reclassification of tumors diagnosed as anaplastic astrocytoma or GBM. Reinhardt et al. [10] performed methylation analysis on a series of 86 tumors initially diagnosed as cerebellar GBM and found that 25 of those met criteria for HGAP. This group also discovered 86 cases of HGAP among 102 tumors initially classified as anaplastic pilocytic astrocytomas [11]. HGAP lacks a distinct histological profile, sharing features with GBM (e.g. microvascular proliferation, high mitotic activity, and necrosis) and pilocytic astrocytoma (e.g. eosinophilic granular bodies and Rosenthal fibers) [3, 10, 11]. Tumors are typically moderately cellular and can range from very circumscribed to focally or, less commonly, diffusely infiltrative; the degree of nuclear atypia is highly variable [11]. Given this constellation of features, it is unsurprising that the prognosis for patients with HGAP lies between that of GBM and pilocytic astrocytoma. This difference in prognosis highlights the importance of performing molecular and epigenetic analyses in order to establish the correct diagnosis as it has implications for patient survival.

Just as no unifying histologic finding characterizes HGAP, this tumor also lacks a consistent radiographic appearance, again occupying a spectrum between GBM and lower-grade tumors. Most of the patients in our cohort had homogenously enhancing lesions, while some had rim enhancing lesions with central necrosis commonly seen in GBM. Other authors have observed primarily rim-enhancing or heterogeneously-enhancing masses [2, 14]. Diffusion restriction is an uncommon finding, though we observed at least a subset of tumors with this radiologic feature [14]. The variable radiologic appearances reported for this entity are likely related to the similarly variable morphologic findings as some tumors are densely cellular whereas others are not, some show exuberant microvascular proliferation, and some demonstrate necrosis. Given this vast spectrum, it is important to remember this diagnostic possibility, both in radiologic and histopathologic differential diagnoses, particularly for tumors located within the posterior fossa.

Importantly, despite the variability in radiologic and histologic appearances, HGAP may be characterized by a distinct set of molecular alterations that can help guide diagnosis, particularly in settings where DNA methylation profiling is not readily available. Alterations in *CDKN2A/B* represent the most common genomic abnormality seen in HGAP, although it is by no means a specific finding and must be taken in conjunction with other radiologic, morphologic, and molecular features [3, 11]. In congruency with previous findings, loss of *CDKN2A/B* were observed in over half of our cohort. *CKDN2A/B* encode proteins p14, p15, and p16, which are involved in regulating cell growth and angiogenesis [16]. Homozygous deletion of *CDKN2A/B* is uniformly associated with a worse prognosis in GBM and IDH-mutant astrocytoma, which may partially explain the aggressive behavior of HGAP [5, 8]. *BRAF* mutations currently carry an uncertain prognostic weight in high grade gliomas, yet they are a significant feature if present as they are a targetable mutation [13]. BRAF inhibitors, which target V600 variants, have been approved for several other malignancies including melanoma and non-small cell lung carcinoma [15]. Two patients in our cohort were treated with BRAF inhibitors at recurrence. One patient was lost to follow up, but the other remains alive at the time of this writing 20 months after diagnosis and 15 months after recurrence. Larger studies with longer-term follow up are needed to determine the utility of such agents for primary or recurrent HGAP. HGAP is also characterized by a high rate of nuclear ATRX loss. [11] Reinhardt et al. [11] observed a 44% rate of nuclear ATRX loss and 23% rate of *ATRX* mutation in their series. While ATRX loss confers radiosensitivity in other high-grade gliomas, no association with survival was observed in HGAP, possibly reflective of the presence of other mutations or young patient age [9, 11]. 

Our findings largely align with existing reports in the literature (see Table 2) [2, 10, 11]. Our patients are mostly middle-aged adults with posterior fossa or thalamic tumors. Mutations in the MAPK pathway, ATRX loss, and *CDKN2A/B* loss were common. While the standard of care for more common glial tumors is well established, the wide variety of treatment courses undergone by patients in our cohort reflects a lack of standardization, which is also evident in the literature. Patients in our cohort most commonly received temozolomide, which is likely to be effective given the reportedly high rate of *MGMT* promotor methylation seen in HGAP, though the majority of tumors in our study set demonstrated absence of *MGMT* promoter methylation [10]. Given the unpredictability in *MGMT* promoter status, it is useful that this tumor often harbors a high rate of other targetable mutations, which presents an opportunity to study alternative agents. Larger series will be needed to determine their utility. The optimal extent of resection for HGAP is also not well-defined. Indeed, other series have not reported data regarding extent of resection [2, 4, 10, 11]. It will be important to define the role of extent of resection in this tumor given its propensity to occur in or near eloquent structures, which explains the low rate of gross total resection observed in our study.

**Table 2:** Existing reports of HGAP

Author (year)	Study/Patients	Surgery	Tumor Locations	Radiation(Y/*N*)	Radiation Dose (Gy)	Systemic Therapy	Progression-free survival	Overall Survival
Goethe et al. (2025)	Single institution (*n* = 18)	Excision (*n* = 9) Biopsy (*n* = 9)	Cerebellum (*n* = 7) Thalamus (*n* = 6) Temporal (*n* = 2) Brainstem (*n* = 2) Spinal cord (*n* = 1)	Y (*n* = 14) N (*n* = 3)	60 (*n* = 4) 54 (*n* = 2) 52 (*n* = 1) 46 (*n* = 1) 40 (*n* = 1) 39 (*n* = 1)	Concurrent + adjuvant TMZ (*n* = 8) Concurrent TMZ (*n* = 3) Adjuvant TMZ (*n* = 2) Trametinib (*n* = 1)	5.4 months	Not reached
Cimino et al. (2023) [4]	Multi institution (*n* = 144)	Excision (*n* = 15) Biopsy (*n* = 14)	*n* = 130 Infratentorial (*n* = 81) Supratentorial (*n* = 34) Spinal cord (*n* = 13)	Not reported	Not reported	Not reported	42.3 months	51.8 months
Reinhardt et al. (2018) [11]	Multi institution (*n* = 83)	Not reported	Infratentorial (*n* = 61) Supratentorial (*n* = 14) Spinal cord (*n* = 6) Supra and infratentorial (*n* = 2)	Not reported	Not reported	Not reported	43.8 months	53.0 months
Bender et al. (2021) [2]	Single institution (*n* = 6)	Excision (*n* = 4) Biopsy (*n* = 2)	Brainstem (*n* = 3) Spinal cord (*n* = 2) Hemispheric (*n* = 1)	*n* = 5 Y (*n* = 4) N (*n* = 1)	*n* = 4 54 (*n* = 2) 50.4 (*n* = 1) 59.2 (*n* = 1)	Concomitant + adjuvant TMZ (*n* = 2)	5.53 months	11.8 months
Soni et al. (2024) [14]	Dual Institution (*n* = 8)	Excision (*n* = 6) Biopsy (*n* = 2)	Brainstem (*n* = 5) Thalamic (*n* = 2) Cerebellum (*n* = 1)	Y (*n* = 6)	Not reported	Not reported	14.5 months	*n* = 6 alive at LFU *n* = 2 died within 1–12 months
Lucas et al. (2022) [7]	Single institution (*n* = 5)	Excision (*n* = 4) Biopsy (*n* = 1)	Brainstem (*n* = 2) Cerebellum (*n* = 2) Thalamus (*n* = 1)	Y (*n* = 4) Unknown (*n* = 1)	Not reported	Trametinib, everolimus (*n* = 1) TMZ (*n* = 1) TMZ + bevacizumab (*n* = 1)	17.1 months	22.6 months
Romo et al. (2023) [12]	Multi institution (*n* = 4)	Excision (*n* = 3) Biopsy (*n* = 1)	Not reported	Y (*n* = 1) N (*n* = 3)	Not reported	Trametinib (*n* = 1) Concomitant TMZ (*n* = 1)	Not reported	8.5 months
Reinhardt et al. (2019) [10]	Multi institution (*n* = 25)	Not reported	Cerebellum (*n* = 25)	Not reported	Not reported	Not reported	Not reported	Not reported
Biczok et al. (2021) [3]	Single institution (*n* = 4)	Biopsy (*n* = 4)	Spinal cord (*n* = 4)	Y (*n* = 4)	Not reported	Yes (*n* = 4), agents not specified	Not reported	8 months (median)
Alturkustani et al. (2023) [1]	Multi institution (m = 1)	Resection	Thalamic (*n* = 1)	Not reported	Not reported	Not reported	31 months	108 months

One of the difficult caveats with the diagnosis of HGAP is grading. Currently, the WHO recommendations do not include an exact tumor grade as this tumor has a broad scale of biologic behavior and therefore it is difficult to precisely characterize it. The waters are further muddied by the historical fact that many such tumors were likely previously diagnosed as “cerebellar GBM” and therefore it is difficult to perform studies involving cases that predate the routine use of molecular study to separate HGAP from bona fide GBM. On one hand, most patients will experience recurrence or regrowth as most cases are incompletely resected simply due to the locations in which these tumors commonly occur. The true biologic behavior surely lies somewhere between a pilocytic astrocytoma and GBM though in reality it leans more toward the behavior of GBM. Thus, as its name states, it should be considered high grade, at least a CNS WHO grade 3, which our data, like that in the literature, support. Time will tell whether this tumor deserves to remain grade 3 or whether it warrants a more aggressive designation of grade 4. These distinctions may be clinically important particularly as it pertains to nomenclature and grading for eligibility for clinical trials; though this tumor is a high-grade glioma, it is its own entity and excludes patients from trials specifically needing a diagnosis of GBM.

The median PFS observed in our study was 5.4 months, which is considerably lower than that of GBM.^8^ We suspect this is not representative of the true behavior of HGAP for several reasons. Firstly, several patients were lost to follow up and received treatment at other institutions, which decreases the sample size, thereby magnifying the significance of short-term survival, and would fail to capture longer survival times. Secondly, our cohort had a high proportion of thalamic and brainstem tumors, which are less amenable to gross total resection. Additionally, several patients did not undergo either chemotherapy or radiation; of those, patients with available follow-up data had poor survival (1.57, 5.23, and 3.17 months). We suspect that further, prospective study with larger cohorts and longer follow-up will demonstrate significantly better survival.

There are several limitations to this study. Due to the rarity and novelty of HGAP and reliance on reclassification of previously obtained specimens, our sample size is relatively small, which limited our analysis. Because many of the patients in our study received care at multiple institutions, loss to follow up was a frequent occurrence and renders meaningful survival analysis difficult. The wide variety of treatments undergone by the patients in this study limits our ability to determine the optimal regimen for HGAP. However, the range of treatments our patients received in a relatively small sample size underscores the importance for continued study of this entity to optimize the most efficacious treatment regimen as we are still in somewhat uncharted territory when it comes to managing these uncommon tumors.

## Conclusion

DNA methylation profiling has recently identified HGAP as a tumor sharing histologic and molecular features with pilocytic astrocytoma and GBM. The unique molecular profile of this tumor presents new opportunities for therapies beyond standard temozolomide-based chemoradiation. Further analysis with longer follow-up and larger series is needed to determine the optimal treatment strategy for this malignancy.

## Data Availability

No datasets were generated or analysed during the current study.

## References

[1] Alturkustani M (2023) Diagnostic insights into pediatric pleomorphic Xanthoastrocytoma through DNA methylation class and pathological diagnosis analysis. Diagnostics (Basel) 13(22):3464. 10.3390/diagnostics13223464 37998600 10.3390/diagnostics13223464 PMC10670667

[2] Bender K, Perez E, Chirica M, Onken J, Kahn J, Brenner W et al (2021) High-grade Astrocytoma with piloid features (HGAP): the Charité experience with a new central nervous system tumor entity. J Neurooncol 153(1):109–120. 10.1007/s11060-021-03749-z 33905054 10.1007/s11060-021-03749-z PMC8131327

[3] Biczok A, Strübing FL, Eder JM, Egensperger R, Schnell O, Zausinger S et al (2021) Molecular diagnostics helps to identify distinct subgroups of spinal Astrocytomas. Acta Neuropathol Commun 9(1):119. 10.1186/s40478-021-01222-6 34193285 10.1186/s40478-021-01222-6 PMC8244211

[4] Cimino PJ, Ketchum C, Turakulov R, Singh O, Abdullaev Z, Giannini C et al (2023) Expanded analysis of high-grade Astrocytoma with piloid features identifies an epigenetically and clinically distinct subtype associated with neurofibromatosis type 1. Acta Neuropathol 145(1):71–82. 10.1007/s00401-022-02513-5 36271929 10.1007/s00401-022-02513-5 PMC9844520

[5] Fortin Ensign SP, Jenkins RB, Giannini C, Sarkaria JN, Galanis E, Kizilbash SH (2023) Translational significance of CDKN2A/B homozygous deletion in isocitrate dehydrogenase-mutant Astrocytoma. Neuro Oncol 25(1):28–36. 10.1093/neuonc/noac205 35973817 10.1093/neuonc/noac205 PMC9825307

[6] Louis DN, Perry A, Wesseling P, Brat DJ, Cree IA, Figarella-Branger D et al (2021) The 2021 WHO classification of tumors of the central nervous system: a summary. Neuro Oncol 23(8):1231–1251. 10.1093/neuonc/noab106 34185076 10.1093/neuonc/noab106 PMC8328013

[7] Lucas CHG, Sloan EA, Gupta R, Wu J, Pratt D, Vasudevan HN et al (2022) Multiplatform molecular analyses refine classification of gliomas arising in patients with neurofibromatosis type 1. Acta Neuropathol 144(4):747–765. 10.1007/s00401-022-02478-5 35945463 10.1007/s00401-022-02478-5 PMC9468105

[8] Ma S, Rudra S, Campian JL, Dahiya S, Dunn GP, Johanns T et al (2020) Prognostic impact of CDKN2A/B deletion, TERT mutation, and EGFR amplification on histological and molecular IDH-wildtype glioblastoma. Neurooncol Adv 2(1):vdaa126. 10.1093/noajnl/vdaa126 33235995 10.1093/noajnl/vdaa126 PMC7668466

[9] Qin T, Mullan B, Ravindran R, Messinger D, Saida R, Cummings JR et al (2022) ATRX loss in glioma results in dysregulation of cell-cycle phase transition and ATM inhibitor radio-sensitization. Cell Rep 38(2). 10.1016/j.celrep.2021.110216 10.1016/j.celrep.2021.110216 PMC8759735 35021084

[10] Reinhardt A, Stichel D, Schrimpf D, Koelsche C, Wefers AK, Ebrahimi A et al (2019) Tumors diagnosed as cerebellar glioblastoma comprise distinct molecular entities. Acta Neuropathol Commun 7(1):163. 10.1186/s40478-019-0801-8 31661039 10.1186/s40478-019-0801-8 PMC6816155

[11] Reinhardt A, Stichel D, Schrimpf D, Sahm F, Korshunov A, Reuss DE et al (2018) Anaplastic Astrocytoma with piloid features, a novel molecular class of IDH wildtype glioma with recurrent MAPK pathway, CDKN2A/B and ATRX alterations. Acta Neuropathol 136(2):273–291. 10.1007/s00401-018-1837-8 29564591 10.1007/s00401-018-1837-8

[12] Romo CG, Piotrowski AF, Campian JL, Diarte J, Rodriguez FJ, Bale TA et al (2023) Clinical, histological, and molecular features of gliomas in adults with neurofibromatosis type 1. Neuro Oncol 25(8):1474–1486. 10.1093/neuonc/noad033 36840626 10.1093/neuonc/noad033 PMC10398805

[13] Sohail A, Virani QUA, Aziz HF, Shamim MS (2024) Prognostic value of BRAF mutation in glioblastoma. J Pak Med Assoc 74(1):185–186. 10.47391/JPMA.24-05 38219188 10.47391/JPMA.24-05

[14] Soni N, Agarwal A, Ajmera P, Mehta P, Gupta V, Vibhute M et al (2024) High-Grade Astrocytoma with piloid features: A dual institutional review of imaging findings of a novel entity. AJNR Am J Neuroradiol 45(4):468–474. 10.3174/ajnr.A8166 38485198 10.3174/ajnr.A8166 PMC11288576

[15] Szklener K, Mazurek M, Wieteska M, Wacławska M, Bilski M, Mańdziuk S (2022) New directions in the therapy of glioblastoma. Cancers (Basel) 14(21):5377. 10.3390/cancers14215377 36358795 10.3390/cancers14215377 PMC9655599

[16] Yuile A, Satgunaseelan L, Wei JQ, Rodriguez M, Back M, Pavlakis N et al (2023) CDKN2A/B homozygous deletions in astrocytomas: A literature review. Curr Issues Mol Biol 45(7):5276–5292. 10.3390/cimb45070335 37504251 10.3390/cimb45070335 PMC10378679

